# Osteoid osteoma in the bones of the hand: a systematic literature review

**DOI:** 10.1007/s00402-023-04839-5

**Published:** 2023-03-20

**Authors:** Jasmin Meyer, Tim Rolvien, Alonja Reiter, Matthias Priemel, Karl-Heinz Frosch, Anna Krukenberg, Sinef Yarar-Schlickewei

**Affiliations:** Department of Trauma and Orthopedic Surgery, University Medical Center Hamburg-Eppendorf, Martinistr. 52, 20251 Hamburg, Germany

**Keywords:** Osteoid osteoma, Hand, Diagnosis, Treatment, Carpus, Phalanges

## Abstract

**Introduction:**

Osteoid osteoma (OO) is a common benign bone tumor. OO is observed most frequently in the long bones, especially in the tibia and femur. When occurring in the bones of the hand, OO can be a diagnostic and therapeutic challenge. The aim of this study was to provide a systematic review of occurrence, symptoms, diagnosis and treatment options regarding OO in hand bones.

**Materials and methods:**

We performed a systematic review of the literature. All studies from the online databases PubMed and SpringerLink, which reported cases of osteoid osteomas in the bones of the hand, were included. By summarizing the literature, we evaluated the localization within the hand as well as diagnostic and therapeutic options.

**Results:**

We included 133 studies reporting 401 cases. OO was mostly common in the phalanges. The diagnosis was mostly made by CT (computed tomography) scan. Most of the OO were treated surgically by open curettage or en bloc resection.

**Conclusions:**

Osteoid osteomas in the bones of the hand are rare and a delayed diagnosis is common. In cases of pain combined with particular symptoms such as nail hypertrophy and swelling OO should be considered. Of the most used imaging methods, CT scans have the highest sensitivity.

## Introduction

Osteoid osteoma (OO), first reported by Jaffe 1935, is a benign bone tumor which is mostly present in the tibia, femur and other long bones, but may also occur at atypical locations [17, 30]. Its typical characteristic in imaging such as radiography, MRI (magnetic resonance imaging) and CT is a central nidus that contains high amounts of osteoid and is surrounded by sclerotic osseous tissue [10, 20]. The etiology of OO is unknown. The tumor is most commonly reported in patients under 30 years of age, with men more likely to be affected [18, 20]. The most typical symptom is nocturnal pain which is primarily sensitive to aspirin, but also other non-steroidal anti-inflammatory drugs (NSAIDs). This is most likely explained by the fact that the nidus inside the osteoid osteoma produces a high amount of prostaglandins [5].

OO in the bones of the hand are rare and, therefore, a diagnostic challenge. Most frequently they occur in the phalanges (59.2%) followed by carpal (30.1%) and metacarpal (10.7%) bones [25]. Many patients with OO have a long history of pain until diagnosis. Radiography and MRI can be less sensitive and lead to misdiagnosis, such as arthritis or cystic lesion [40, 43]. OO in the distal phalanges can also lead to deformity with hypertrophy of the nailbed [23, 36]. Due to the frequent proximity to joints and many other relevant structures in the hand as well as the small size of the bones, there may be difficulties in the treatment of OO in the hand. An overview of diagnostic and therapeutic options appears to be important to improve the treatment of the tumor.

The purpose of this systematic review was to provide a thorough and detailed assessment of reported cases of OO in the bones of the hand. The objectives of this article were to analyze patient age, symptoms, the time to diagnosis, the sensitivity of the used diagnostic modalities, the frequency of the effected bones and the conducted treatments.

## Methods

### Search strategy

The original protocol for this systematic literature review was registered on PROSPERO, the international prospective register of systematic reviews, which can be assessed online without any limitations (CRD42020221735) [7]. The Database of Abstracts of Reviews of Effects, the Cochrane Database of Systematic Reviews, and PROSPERO could not identify any previously performed systematic reviews investigating OO in hand.

The following literature search was done according to the reporting item for systematic reviews PRISMA [27]. Two major databases PubMed and SpringerLink were searched independently by two reviewers. Both medical databases were searched from inception through December 1, 2020. A complementary search was also performed, which included review of bibliographies of articles of interest [3]. There were no limitations on date of publication or type of journal.

The used search algorithm was: ["osteoid osteoma"] OR ["osteoid osteoma" [MeSH Terms]] AND [hand OR "radial joint" OR scaphoideum OR scaphoid OR lunatum OR capitatum OR triquetrum OR hamatum OR pisiforme OR trapezoideum OR trapezium OR phalangeal OR phalangen OR finger OR metacarpal].

Only articles written in English were included. Inclusion criteria were clinical studies including case reports or case series reporting on OO in the bones of the hand. The following criteria was used for exclusion: (1) cadaveric study, (2) wrong subject (e.g., no OO), (3) review papers without their own cases, (4) OO in localization other than hand, (5) no English pdf, (6) localization not detected, (7) publications reporting on OO in animals.

**Fig. 1:**
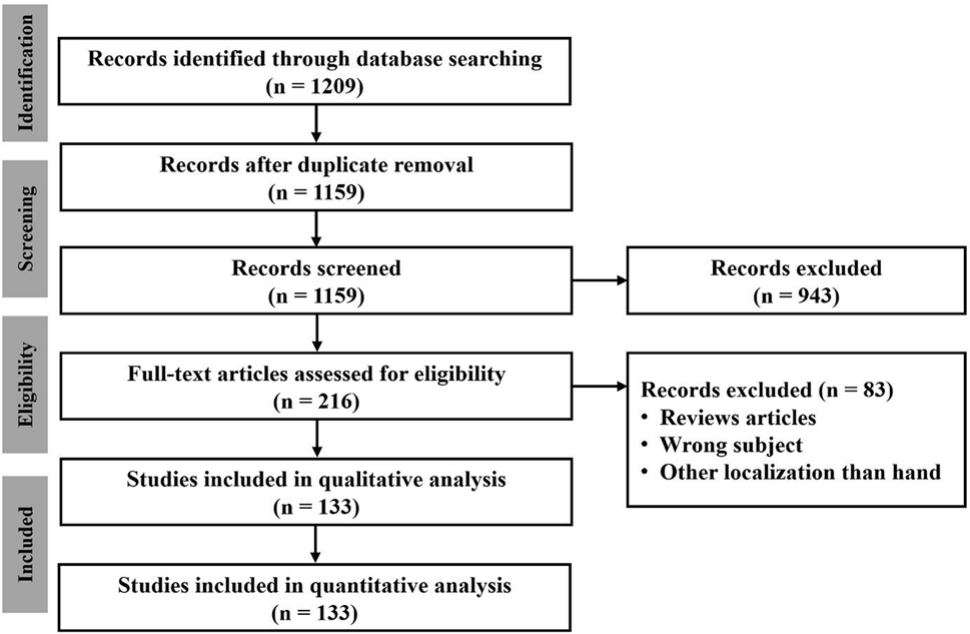
Flow chart to display the review process. The literature search was carried out using the PRISMA guidelines (Preferred Reporting Items for Systematic Reviews and Meta Analyses)

Two experienced reviewers independently screened titles and abstracts for the study selection process. Any disagreements were discussed and final decisions were made based on group consensus. 216 manuscripts were included for full test review (Fig. 1).

### Data extraction

The following data were extracted from each reported case when stated: patient age; gender; affected bone; cancellous, intracortical or subperiosteal localization; pain; nocturnal pain; decrease of pain under NSAID; swelling; deformity; reduced motion in the along sided joints; used image modalities (radiography, MRI, CT, SPECT (single photon emission computed tomography), bone scintigraphy) and their sensitivity; results of the histopathological analysis; time to diagnosis; treatment; time to follow up; complications. Data extraction was performed independently by two observers.

### Quality assessment

A quality assessment was carried out on all included studies independently by two reviewers using the methodological index for non-randomized studies (MINORS) [35].

### Statistical and data analysis

Descriptive statistics were utilized for analysis of included studies. The mean values of applicable numerical data within the included reports were pooled, calculated, and presented. IBM SPSS Statistics version 26.0 (IBM, Armonk, NY) was used for statistical analysis.

## Results

### Search results and study characteristics

**Fig. 2:**
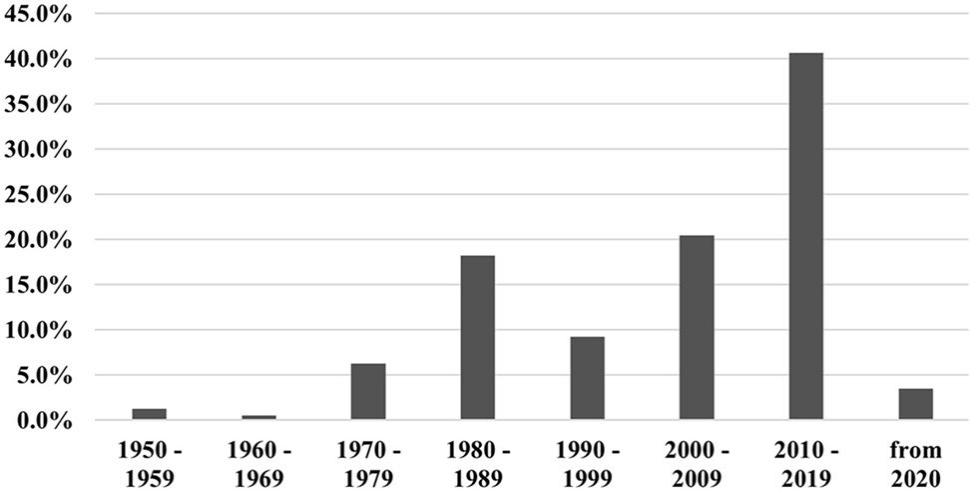
Year of publication of the included studies

After exclusion of duplicates and implementation of inclusion and exclusion criteria, 133 manuscripts were reviewed. In total, the data of 401 OO cases in the bones of the hand were extracted. Most of the manuscripts were case reports (*n* = 90), 43 were case series with a pooled mean of 9.3 (range 2–37) included cases. The studies were mainly published between 2010 and 2020 (Fig. 2). We only included data regarding localization, symptoms and treatment that were explicitly stated in the manuscript.

### Epidemiology

**Fig. 3:**
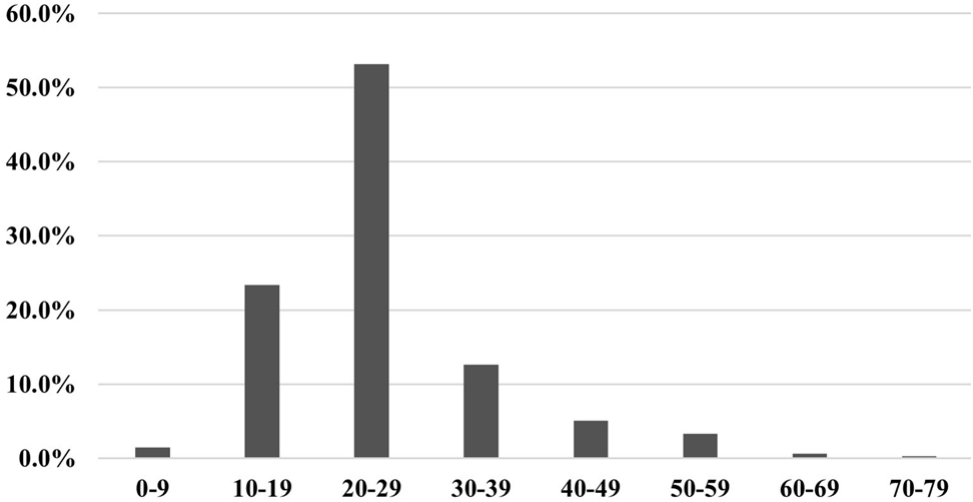
Age of the included patients

In 356 of the 401 reviewed cases, the gender of the patients was mentioned. From these 356 cases, 219 (61.5%) were males and 137 (38.5%) females. Most of the patients were aged between 20 and 29 (Fig. 3). The mean pooled age was 26 (standard deviation = 10.1).

**Fig. 4:**
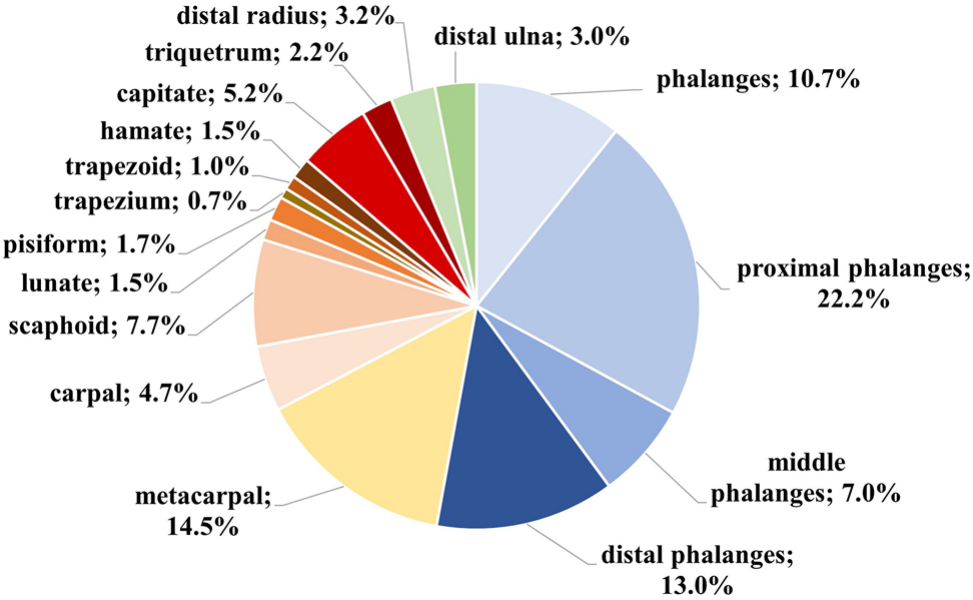
Distribution of described osteoid osteoma in different affected bones of the hand

Regarding the localization of OO within the hand, the phalanges were the most affected bones (52.9%). In particular, OO most commonly occurred in the proximal phalanges (22.2%) followed by the metacarpals (14.5%) and the distal phalanges (13.0%). The scaphoid was the most frequently affected carpal bone (7.7%). A detailed summary of the affected bones is shown in Fig. 4 (Fig. 4). The localization of OO in the hand bones (i.e., intracortical, cancellous, subperiosteal, juxta-articular) was described only in a few cases (*n* = 37). It was mainly localized intracortical (46.0%; *n* = 17), followed by cancellous (29.7%; *n* = 11), subperiosteal (16.2%; *n* = 6) and juxta-articular appearance (8.1%; *n* = 3).

### Symptoms and time to diagnosis

The mean pooled time to diagnosis was 19 months with a range from 2 months to 25 years. In most cases, pain was mentioned as the most common symptom. Namely, pain was reported in 95.1% of the cases. There was no information regarding pain as a symptom in 135 of the cases. An increase in pain or especially pain at night was described in 90.4% of the cases. 84.2% of the patients described an improvement in pain due to NSAID. Especially in the phalanges, swelling was described as a heaped symptom (99.0%). A peculiarity of OO in the distal phalanges was the frequent occurrence of deformities, such as hypertrophy of the nail bed (*n* = 36). Whether restrictions on mobility existed was rarely discussed (*n* = 87). In these cases, about half of the patients were affected by restrictions on mobility (Table 1).

**Table 1:** Summary of symptoms of patients with osteoid osteoma reported in 290 cases

	Pain (%)	Night pain (%)	NSAIR response (%)	Swelling (%)	Deformity (%)	Reduced motion (%)
All patients	95.1	90.4	84.2	97.2	86.4	50.6
Proximal phalanges	88.9	83.9	87.8	98.0	2.2	70.8
Middle phalanges	85.7	83.3	44.4	100.0	0.0	0.0
Distal phalanges	95.6	96.7	95.8	100.0	69.2	71.4
Phalanges	88.9			99.0	0.0	0.0
Metacarpal	100.0	92.9	80.0	92.3	0.0	66.7
Scaphoid	100.0	92.9	81.3	100.0	0.0	62.5
Lunate	100.0	100.0	100.0	100.0	0.0	0.0
Pisiform	100.0	100.0	100.0	80.0	0.0	50.0
Trapezium	100.0	100.0	100.0		0.0	
Trapezoid	100.0	0.0	66.7		0.0	
Hamate	100.0	100.0	100.0	100.0	0.0	100.0
Capitate	100.0	83.3	81.8	85.7	0.0	80.0
Triquetrum	100.0	100.0	60.0	100.0	0.0	66.7
Carpal	100.0				0.0	25.0
Distal radius	100.0	100.0	100.0	100.0	0.0	
Distal ulna	100.0		100.0		0.0	

### Imaging modalities

X-rays and CT scans were the most used imaging modalities followed by MRI and scintigraphy. Individual studies used SPECT as complementary imaging (*n* = 12). Importantly, of the most used imaging methods, CT showed the highest sensitivity (93.1%) followed by MRI (81.6%) (Table 2).

**Table 2:** Sensitivity of the used imaging modalities for diagnosis of osteoid osteoma reported in 242 cases

	X-ray (%)	CT (%)	MRI (%)	Szintigraphie (%)	SPECT (%)
All patients	64.4	93.1	81.6	90.5	100.0
Proximal phalanges	73.1	96.9	93.1	89.5	100.0
Middle phalanges	68.4	100.0	100.0	80.0	100.0
Distal phalanges	76.7	100.0	80.0	100.0	
Phalanges	100.0				
Metacarpal	60.9	95.8	92.3	91.7	100.0
Scaphoid	58.8	94.1	72.7	100.0	100.0
Lunate	20.0	100.0	100.0	66.7	
Pisiform	28.6	100.0	0.0		100.0
Trapezium	0.0	100.0	100.0		
Trapezoid	50.0	100.0	100.0	100.0	
Hamate	50.0	0.0	50.0	100.0	
Capitate	61.5	66.7	66.7	50.0	
Triquetrum	75.0	75.0	33.3	100.0	
Carpal					
Distal radius	0.0	66.7	50.0		
Distal ulna	100.0	100.0			

### Treatment, follow-up and complications

Most of the included cases (*n* = 205) were treated surgically. In 164 cases, no further details were reported about the therapy. Of the 237 included patients, 42.2% (*n* = 100) were treated with en bloc resection and 40.5% (*n* = 96) with open curettage. Other less commonly used treatments were laser photocoagulation (6.3%; *n* = 15), thermocoagulation (5.1%; *n* = 12) and radiofrequency ablation (3.8%; *n* = 9). Amputation was used in 1.7% (*n* = 4). Only one case was treated conservatively. In 122 cases the results of a histopathological examination were provided. Of these, 120 were diagnosed positively with OO. In two patients the primary pathological examination was negative and the OO was histologically secured in a subsequent sample. According to this, the primary pathological examination in the cohort studied showed a sensitivity of 98.4%. The mean follow-up was 39 months with a range from 1 month to 11 years. With a total of 34 cases, only a few complications were described. The most common complication was recurrence (85.3%) followed by infection (5.9%) and persistent pain (5.9%). One case reported postoperative motor deficits (2.9%). Most of the recurrences occurred after open curettage (*n* = 10), followed by en bloc resection (*n* = 8) and thermocoagulation (*n* = 5). In six cases there was no further information related to previous therapy.

### Quality assessment

All 133 studies were included in a quality assessment using the validated methodological index MINORS. The average score was 8 (range 3–11), with the global ideal score being 16 for non-comparative studies and 24 for comparative studies.

## Discussion

This article provides a systematic overview and detailed analysis of epidemiology, symptoms, diagnostic and treatment of OO in the hand bones. In the literature there is a frequent occurrence of OO in the second and third decade [2, 5, 18]. The average age in the cohort studied was 26 years, with most patients aged between 20 and 29 years (53%). A frequent occurrence of OO in male patients could also be confirmed in our review [10, 34]. Together, these epidemiologic data are consistent with the literature, although this review shows that relatively more women may be affected and the mean age is slightly higher (female 38.5%, male 71.1%) [30]. When examining the most affected bones, there is an increased incidence in the proximal phalanges. As expected, the appearance of OO in the carpal bones was less frequent.

OO can be localized in the cancellous (i.e., trabecular) or cortical bone, with special localizations, such as subperiosteal or juxta-articular (i.e., intra-articular) [8, 20]. In the hand, a closer look at the localization within a bone is interesting, since in particular the carpal bones are small, covered with cartilage and a joint-near position of the OO is probable. The localization of the lesion has only been described in 37 of the reviewed cases. As described in the literature, intracortical OO was most common [20, 33]. Subperiosteal localization, rarely reported in the hand bones, is less common and can be a diagnostic challenge when occurring juxta-articular [11, 12, 37]. Juxta-articular localization of OO can lead to joint pain mimicking monoarthritis, which is why these cases are often misdiagnosed [31]. The nidus, which is surrounded by sclerotic bone, is also harder to identify in imaging caused by the complex radiological anatomy of the joint [14]. In addition, OO can be masked by a marrow edema [31]. Six cases of subperiosteal and three cases of juxta-articular localization were reviewed. We did not observe a frequent occurrence of pain radiation or mobility restrictions in the adjacent joints.

Local pain that responds to NSAID and increases at night is considered a typical symptom of OO [5] [16]. In our review, local pain was confirmed as a leading symptom in more than 95% of the cases. In most cases, there was additional pain at night and an improvement was achieved by NSAID. The combination of nocturnal pain sensitive to NSAIDs in combination with a typical lesion in imaging should be indicative of OO [31]. A symptom that has been described particularly frequently in OO in the proximal phalanges is local swelling. In some cases, the swelling occurred before the onset of pain [6, 38, 44]. In many of the reviewed cases OO in the distal phalanges led to deformity with hypertrophy of the nailbed [1, 23, 36, 43]. Levy et al. also reported a case of a 14-year-old female with OO in the distal phalanx of the thumb causing epiphysial growth arrest [23]. Limitations in the mobility of the surrounding joints were described less frequently. In some cases, the symptoms, such as pain and swelling, radiated into the surrounding joints [6, 19, 29]. Many patients with OO have a long history of pain until diagnosis [1, 29, 43]. With an average delay until diagnosis of 19 months, this statement can be confirmed in our review. These results are consistent with the experiences of patients with OO treated in our department of hand surgery. Most patients have been describing complaints for several months. In addition, some have been treated with suspected diagnoses, such as overuse or arthritis. In most cases, a nidus visible on CT scan led to the correct diagnosis.

CT scans have a high sensitivity in identifying OO which typically show a central nidus surrounded by sclerosis [24]. Of all the imaging modalities used in the reviewed cases the CT showed the highest sensitivity with 93.1%. Thin-section CT scans should be used when OO is suggested in the bones of the hand [4]. CT scans also allow visualization and precise localization of OO and are, therefore, helpful for preoperative planning [15]. Plain Radiography and MRI can be less sensitive and can lead to misdiagnosis, such as arthritis or cystic lesion [40, 43]. X-ray, which is often performed to diagnose hand complaints, had the lowest sensitivity at 64.4%. In many cases, the typical imaging characteristic of OO were revealed in X-rays well after the onset of the symptoms [6, 13, 43]. MRI is often used when patients have a long history of pain in the hand combined with swelling and tenderness. The sensitivity of MRI in the cohort studied was slightly higher at 81.6% than stated in the literature in localizations other than the hand [9, 18]. In MRI imaging, OO is described as a visible nidus surrounded by reactive marrow edema within the affected bone [21, 28]. Encircling the bone there can be an increase of soft tissue mass [21]. Scintigraphy was often performed as complementary imaging and had a high sensitivity of 90.5%. Scintigraphy using Technetium shows a diffuse increased uptake in the area of the OO [15]. Because of the symptoms typical for OO (pain, swelling, tenderness) an increased uptake can also be misdiagnosed as painful monoarthritis [21]. Accordingly, Scintigraphy has a status as a supplementary imaging to the CT or MRI to avoid misdiagnosis. SPECT has been used rarely and the case number is too small to make a valid recommendation.

Most of the OO cases in the bones of the hand are treated surgically with en bloc excision or open curettage [15, 21]. Importantly, surgical treatment offers the advantage of histological confirmation. Difficulties arise due to the small diameter of the bones and the proximity to important neurovascular structures. Bone defects can be filled with autogenous cancellous bone, which can be obtained from the iliac crest or the distal radius [15, 21]. Recurrences are rare. In OO in the distal phalanges, a correction of deformities may be performed simultaneously. In our department of hand surgery, we have had good experiences with the surgical removal of OO performing an open curettage. Larger defects were filled with cancellous bone from the distal radius.

In recent studies, percutaneous radiofrequency ablation (RFA) under computed tomography guidance is used as an alternative treatment for OO in the hand [26, 39]. It leads to good outcomes and a low morbidity [39]. When used for lesions in the hand and feet RFA must be used with caution as there is a risk of subsequent osteonecrosis or thermal damage to adjacent neurovascular structures [22, 30, 32]. When treating OO by RFA, it is important to reach inside the nidus to eliminate the lesion entirely [39]. Similar results are described in a study which used CT guided percutaneous laser photocoagulation to treat 15 cases of OO of the hands and feet [45]. Important are the placement of the optical fiber in the center of the nidus and the adaptation of the energy dose to the size of the nidus. Some studies used thermocoagulation to treat OO in the hand with a low morbidity after procedure but a high recurrence rate [42].

Overall, only little complications have been described after treatment, which may be due to the fact that OO are benign tumors with a small diameter [41]. From all complications, recurrence has been most commonly reported [32]. Most recurrences occurred after thermocoagulation (5 out of 12 cases) followed by open curettage (10 out of 96 cases) and en bloc resection (8 out of 100 cases). There were only a few cases of OO treated by laser photocoagulation or radiofrequency with no complications described in the follow-up examination.

Some limitations should be noted regarding this review. As with any systematic literature review, this study was directly limited by the quality of included literature. The majority of included source literature was case reports and case series with an evidence level of 4 or 5. This is also reflected in the results of the Quality assessment with a low average score. Only information that was explicitly described in the studies could be collected. Valid statements on selected questions such as the quality of the pain and the localization of OO in the bone could not be made due to the lack of data. As a limitation of the presented review, it should be noted that rare cases are more likely to be published, which could affect the distribution of the affected bones. Regarding the surgical therapy of OO, additional information on access routes, difficulties and post-treatment schemes would be useful. To determine the number of complications, especially of recurrences, a longer time to follow up in some of the case reports and case series would be necessary.

## Conclusion

We reviewed 401 cases of OO in the bones of the hand. OO was most common in the proximal phalanges followed by the metacarpals and the distal phalanges. The frequency of typical symptoms such as pain, night pain and response to NSAIDs could be confirmed in the reviewed cases. Furthermore, if swelling and nail hypertrophy occurs, the diagnosis of OO in the phalanges should be considered. In some cases, motor limitations of the surrounding joints have been described. Together, the time to diagnosis is often prolonged in patients with OO in the bones of the hand. CT has the highest sensitivity and should be performed in case of suspicion of an OO. Most OO on the hand are surgically treated with excellent results. In recent years, studies with alternative therapies such as radiofrequency ablation, laser photocoagulation and thermocoagulation have also been shown to be effective.

## Data Availability

The data that support the findings of the study are available from the corresponding author, [Meyer, Jasmin], upon reasonable request.
